# A Novel NLRP3 Inhibitor AMS‐17 Rescues Deficits in Long‐Term Potentiation Following Mild Traumatic Brain Injury in Adult C57Bl/6 Mice

**DOI:** 10.1002/hipo.70072

**Published:** 2026-01-18

**Authors:** Eric Eyolfson, Luis Bettio, Justin Brand, Naveen Kumar Gupta, Emily Hamer, Ryan Salas, Amol Kulkarni, Brian R. Christie

**Affiliations:** ^1^ School of Medical Sciences University of Victoria Victoria Canada; ^2^ Department of Chemistry The University of Texas at El Paso El Paso Texas USA; ^3^ Department of Psychology San Diego State University San Diego California USA; ^4^ Island Medical Program and Department of Cellular and Physiological Sciences University of British Columbia Victoria Canada

**Keywords:** dentate gyrus, electrophysiology, hippocampus, long term potentiation, medial perforant pathway

## Abstract

Traumatic brain injury (TBI) is a leading cause of long‐term disability, with limited effective treatment options. A key factor of TBI pathophysiology is neuroinflammation, which can involve the activation of the nucleotide‐binding domain leucine‐rich repeat protein 3 (NLRP3) inflammasome. Aberrant inflammation following injury has the ability to reduce the capacity to induce long‐term changes in synaptic plasticity, a leading mechanism for the development of learning and memory deficits following injury. This study investigated the potential of a novel NLRP3 inhibitor, AMS‐17, to mitigate synaptic plasticity deficits following mild TBI (mTBI) in mice. Adult C57Bl/6 mice were subjected to mTBI or a sham injury, and hippocampal slices were then prepared for field electrophysiological recordings in the medial perforant pathway of the dentate gyrus. We found that mTBI induced deficits in long‐term potentiation that were not immediate at 2 h post‐injury but developed by 3 days post‐injury. We next incubated slices in AMS‐17 or a control solution prior to electrophysiological recordings. Here we found that incubation with AMS‐17 rescued these LTP deficits, bringing them to levels observed in sham‐injured controls. Importantly, AMS‐17 did not affect the capacity to induce LTP in sham‐injured mice. These findings suggest that targeting the NLRP3 inflammasome may offer a promising therapeutic strategy to reduce learning and memory impairments following mTBI. Further studies are needed to determine the optimal therapeutic window and long‐term efficacy of AMS‐17 in mTBI.

## 1 Introduction

Traumatic brain injury (TBI) is a term used to refer to a subset of brain injuries that can be induced by the application of external mechanical forces to the head, neck, or body (Sharp and Jenkins 2015). The majority of all TBI's are defined as mild TBI (mTBI), yet despite this “mild” designation, mTBI can result in serious, long‐lasting consequences, including prolonged learning and memory impairments (Barlow et al. 2010; Patricios et al. 2023). Indeed, mTBI is reported to be one of the leading causes of long‐term disability and is accompanied by significant personal and economic burden (Fu et al. 2016; Gaudette et al. 2022). Unfortunately, current treatment options are often ineffective, poorly tolerated, and have not significantly improved long‐term neurological outcomes. Thus, there is growing interest in exploring novel therapeutic targets for managing mTBI.

A mTBI, whether in humans or animals, consists of both a primary and secondary injury phase. The primary phase is the direct result of the external forces causing the brain to be subjected to rotational acceleration and deceleration, which can result in diffuse axonal injury (Mckee and Daneshvar 2015). The secondary injury cascade consists of an initial inflammatory response that is essential for recovery, however it can also be accompanied by prolonged and dysregulated neuroinflammation that can lead to neurodegeneration and persistent deficits (Simon et al. 2017). A crucial mediator of TBI‐associated inflammation is the nucleotide‐binding domain leucine‐rich repeat protein 3 (NLRP3) inflammasome (O'Brien et al. 2020). Pre‐clinical models have shown that NLRP3 activation can result in maturation of interleukin‐1 beta (IL‐1 β), increased microglial reactivity, and neuronal injury (Cai et al. 2022; Jha et al. 2010; Martinon et al. 2002). Importantly, small‐molecule inhibitors of NLRP3 (i.e., JC124; MCC950) have shown beneficial effects in pre‐clinical models of moderate‐to‐severe TBI. Including reduced hippocampal neurodegeneration, lesion volume, proinflammatory profile, as well as improving cognitive deficits (Xu et al. 2018; Kuwar et al. 2019).

The leading candidate for the neural processes behind learning and memory is considered to be synaptic plasticity. All severities of TBI demonstrate the ability to induce long‐term potentiation (LTP) deficits in the hippocampus. In mTBI, these deficits emerge as early as post‐injury day 1 (PID1) and can persist through PID30 (Almeida‐Suhett et al. 2015; Liu et al. 2017; White et al. 2017; Lecca et al. 2019; Sloley et al. 2021; Hu et al. 2022). Unfortunately, the majority of preclinical studies have focused on the CA1 region of the hippocampus leaving the dentate gyrus (DG) understudied. This is critical as the DG is the beginning of the tri‐synaptic hippocampal circuit and is one of the few sites of adult neurogenesis (Eadie et al. 2005; Christie and Cameron 2006; Ernst and Christie 2006). Previous studies have demonstrated that mTBI transiently increases cellular proliferation in the hippocampus that resolves within 1 week of injury (Dash et al. 2001; Sun et al. 2005; Neale et al. 2023). Importantly, a significant proportion of these proliferating cells have been associated with astrocytes and microglia. This suggests that the DG may be uniquely vulnerable to an inflammatory response compared to the CA1. Mechanistically, inflammation and plasticity converge through activation of the NLRP3 inflammasome. Previous reports suggest that NLRP3 activation promotes caspase‐1‐dependent maturation of IL‐1 β (Martinon et al. 2002) which can in‐turn modulate N‐methyl‐D‐aspartate receptor (NMDAR) signaling (Viviani et al. 2003; Yang et al. 2005; Gardoni et al. 2011), and impair the induction of LTP (Coogan et al. 1999; Curran and O'Connor 2001).

Unfortunately, little is known about the effects of NLRP3 inhibition following closed‐head mTBI, resulting in a critical gap in knowledge given mTBI are the most common form of TBI. Despite encouraging findings in moderate‐to‐severe TBI, the use of current NLRP3 inhibitors have significant translational challenges and most have yet to be approved for clinical application (Xu et al. 2025). For example, MCC950 is a secondary sulfonylurea compound with potent NLRP3 inhibitory activity and displays protective activity in mice against TBI (Ismael et al. 2018). However, MCC950 presents a variety of adverse effects, including severe hepatotoxicity (Charan et al. 2023), and off‐target binding to carbonic anhydrase‐2 (Kennedy et al. 2021). These adverse effects may limit the use of MCC950 for the treatment of TBI. Thus, the development of an effective, safe therapy for the treatment of TBI remains an urgent and unmet challenge. Recently a novel sulfonylurea‐derived compound, AMS‐17, has shown the ability to supress IL‐1 β protein and gene expression in microglial cell cultures and reduce the density of Iba1+ microglia in the mouse cerebral cortex following lipopolysaccharide (LPS) injections (Zhang et al. 2022). Therefore, the goal of the current study was to build upon these results and investigate the therapeutic potential of AMS‐17 to recover LTP deficits following mTBI in mice.

## 2 Methods

### 2.1 Animals

Female and male C57Bl/6 mice were bred in house at the University of Victoria, Canada. Animals were adults (10–12 weeks) at the time of mTBI. Mice were housed in groups (*N* = 4–5) in transparent polycarbonate cages and had ad libitum access to food and water and maintained on a 12:12 light/dark cycle (lights on at 06:00). The housing rooms were kept at a constant temperature (22°C–26°C) and humidity (40%–60%). Mice were kept on a standard diet of rat chow. Protocols were approved by the University of Victoria animal care committee and in accordance with Canadian Council of Animal Care guidelines and standards.

### 2.2 Mild Traumatic Brain Injury

Injuries were administered using a Gothenburg impactor (Collision Analysis, Calgary, Canada) (Viano et al. 2009). In brief, adult mice were anesthetized with 5% isoflurane in a VetFlo‐0530SM induction chamber (Kent Scientific, Torrington, CT, USA) and placed in a prone position on a Teflon board. A 50‐g weight was propelled (7.03 ± 0.05 ms) toward an aluminum “helmet” (W:35 × H:14 × D:3 mm) that was affixed to the side of the impactor to transfer force to the mouse and prevent skull fractures (Mychasiuk et al. 2016). Mice then underwent 180° horizontal acceleration/deceleration. Sham procedures followed the same protocol with the exception that the device was not triggered. Following the mTBI or sham injury, mice were placed in a supine position on a warming pad (32°C; Kent Scientific, Torrington, CT, USA) in a clean cage and measured for their time to flip into a prone position. Time‐to‐right was used as a measure of loss of consciousness (Eyolfson et al. 2020, 2022).

### 2.3 Slice Electrophysiology

Mice were euthanized for electrophysiological recordings 2 h or 3 days following the mTBI or sham injury as described in (Petersen et al. 2013; White et al. 2017; Christie et al. 2023). In brief, mice were anesthetized with inhaled isoflurane and quickly decapitated. Brains were rapidly removed and prepared for sectioning in chilled (4°C) artificial cerebrospinal fluid (aCSF; in mM): 125 NaCl, 3.0 KCl, 1.25 NaH_2_PO_4_, 25 NaHCO_3_, 2 CaCl_2_, 1.3 MgCl_2_, and 10.4 dextrose, and continuously bubbled with carbogen (95% O_2_/5% CO_2_) and adjusted to a pH between 7.3 and 7.4. Transverse (350 μm) slices were sectioned on a Compresstome 310‐0Z (Precisionary, Ashland, MA, USA). After sectioning, slices were allowed to recover in carbogenated aCSF in a water bath for at least 60 min at 32°C before being maintained at room temperature for the remainder of the experiment.

Hippocampal slices were transferred to a PC‐V recording chamber (Siskiyou, Grants Pass, OR, USA) and visualized at 4× magnification with an upright (Olympus BX51WI, Olympus, Center Valley, PA, USA). The medial perforant pathway (MPP) of the DG was visually identified and a concentric bipolar simulating electrode (FHC, Bowdinham, ME, USA) and a glass recording micropipette (1–2 mΩ resistance) filled with aCSF were visually placed approximately 200 μm apart in the MPP. The MPP was defined as the middle third of the DG, approximately 100 μm above the molecular layer. Field excitatory postsynaptic potentials (fEPSPs) were evoked (0.12 ms; once every 15 s) and recorded with an Axon Multiclamp 700B amplifier, digitized by an Axon Digidata 1550A, and recorded using Clampex 10.5 software (Molecular Devices, San Jose, CA, USA). Electrode placements were adjusted to evoke an fEPSP with a minimum amplitude of 0.70 mV. The response size was adjusted to 50%–60% of the maximum response size (average stimulation: 0.04 ± 0.01 mA). This response size was then used for the rest of the experiment. Presynaptic neurotransmitter released was then assessed using paired‐pulse stimuli (6 pairs of pulses 50 ms apart). A paired‐pulse ratio was calculated by taking the slope of the second pulse divided by the slope of the first pulse to ensure that significant facilitation, indicative of lateral perforant path activation, was not produced (Petersen et al. 2013). A series of 10 stepwise pulse widths from 30 to 300 μs was then administered to establish input/output (I/O) curve for each slice (Kannangara et al. 2015).

To facilitate the induction of long‐term potentiation (LTP) in the DG, the GABA‐A antagonist (bicuculine methiodide; 5 μM) was included in the aCSF prior to and during the application of High Frequency Stimulation (HFS; 4 × 100 Hz; 0.24 μs; 30 s inter‐train interval) (Eyolfson, Acosta, et al. 2024). Prior to the application of HFS conditioning stimuli, a baseline was established with single pulse stimulation (1 pulse every 15 s; 0.067 Hz) for a minimum of 15 min. Following the application of HFS, single pulse stimulation was again resumed for 60 min. Post‐conditioning slope of fEPSP signals were averaged into 1‐min intervals (4 sweeps) and calculated as a percent change from the pre‐conditioning recordings. All data is shown as the mean ± standard error of the mean.

### 2.4 Drugs

A subset of hippocampal slices was incubated in 2.8 μM AMS‐17 in regular aCSF from slicing until recordings took place (1–4 h). Control slices were incubated with regular aCSF.

#### 2.4.1 Synthesis of AMS‐17

**FIGURE 1:**
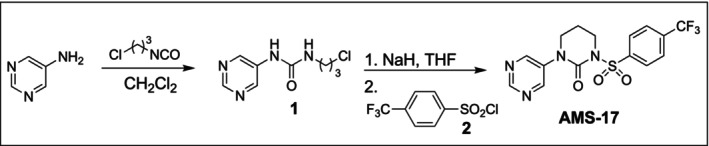
Schematic of the synthesis of AMS‐17.

The synthesis of AMS‐17 is described in Figure 1. All the reagents and solvents were purchased from commercial sources such as Sigma‐Aldrich and Oakwood Chemical, unless otherwise specified. Chemical reactions were monitored using thin‐layer chromatography (TLC) on Whatmann silica gel 60 Å plates with a fluorescent indicator and visualized using a UV lamp (254 nm). Purification of compounds was carried out using column chromatography with Merck silica‐gel (60–120 mesh) as the stationary phase. ^1^H NMR and ^13^C NMR data were recorded on a Bruker AVANCE III (^1^H 400 MHz, ^13^C 100 MHz) spectrometer with CDCl_3_ or DMSO‐*d*_6_ as solvents. ^1^H chemical shifts were referenced to CDCl_3_ at 7.26 ppm and for DMSO‐*d*_6_ at 2.50 and 3.31 ppm. ^13^C chemical shifts were referenced to CDCl_3_ at 77 ppm and DMSO‐*d*_6_ at 39.8 ppm and obtained with ^1^H decoupling.

##### 2.4.1.1 1‐(3‐Chloropropyl)‐3‐(Pyrimidin‐5‐yl)Urea (**1**)

In a 10 mL round bottle flask, 5‐Aminopyrimidine (48 mg, 0.5 mmol, 1.0 equiv.) was dissolved in dichloromethane (4 mL) at room temperature (25°C), and then 3‐chloropropyl isocyanate (66 mg, 0.55 mmole, 1.1 equiv) was added under an argon atmosphere. The reaction mixture was stirred at ambient temperature for 72 h. The reaction was monitored by thin layer chromatography (TLC). After completion of the reaction, the volatiles were removed under reduced pressure, and the resultant crude product was purified by silica gel column chromatography. Elution with ethyl acetate: hexanes resulted in the isolation of the desired product 1 (99 mg, 92% yield) as a pale yellow solid. ^1^H, ^13^C‐NMR, and FT‐IR spectra for acyclic urea **1** matched the previous report (Zhang et al. 2022).

##### 2.4.1.2 1‐(Pyrimidin‐5‐yl)‐3‐((4‐(Trifluoromethyl)Phenyl)Sulfonyl)Tetrahydropyrimidin‐2 (1H)‐One (AMS‐17, 3)

Acyclic urea **1** (107 mg, 0.5 mmole, 1 equiv.) was dissolved in anhydrous tetrahydrofuran (4 mL) and cooled to 0°C by using an ice bath. NaH (60 mg, 3.0 equiv., 60% suspension in paraffin oil) was slowly added. The reaction mixture was stirred at 0°C for 30 min then at room temperature overnight. Sulfonyl chloride **2** (134 mg, 0.55 mmole, 1.1 equiv.) was added to the solution. The reaction was stirred at ambient temperature for 16 h. The crude was purified by silica gel column chromatography. Elution with ethyl acetate: hexanes resulted in the isolation of the desired product (146 mg, 76% yield) as a colorless solid. ^1^H, ^13^C‐NMR, and FT‐IR spectra for acyclic urea 1 matched the previous report (Zhang et al. 2022).

### 2.5 Statistics

Electrophysiological data was analyzed in Clampfit 11.2 (Axon Instruments, Molecular Devices, San Jose, CA, USA). For electrophysiological data, the number included in the final sample is shown as both the N for animals, as well as the n for slices: Sham + aCSF (*N* = 8; *n* = 13), Sham + AMS‐17 (*N* = 4; *n* = 13), mTBI + aCSF (*N* = 9; *n* = 17), and mTBI + AMS‐17 (*N* = 4; *n* = 12). There were no significant differences (*t*(15) = 2.13, *p* = 0.67) between female and male LTP response to mTBI, so data was collapsed across sex for ease of interpretation. Power calculations were run in G*Power (Version 4) with a moderate effect size of 0.4, alpha of 0.05, and power of 0.8, using four groups resulted in a projected sample size of 52. Thus, we were adequately powered for electrophysiological comparisons between compound and injury conditions. Electrophysiology data passed checks for normality (Shapiro–Wilk; *p*‐values > 0.05) and homogeneity of variance (Levene's test; *p*‐values > 0.05). Statistics were run in SPSS version 31 for macOS (SPSS Inc., Chicago IL, USA). To initially compare the effect of mTBI on LTP we ran an unpaired *t*‐test for injury (Sham; mTBI). We ran a two‐way ANOVA for injury (Sham; mTBI) and incubation drug (aCSF; AMS‐17). A repeated measures ANOVA was used to analyze within (pulse width) and between‐subjects (injury and compound) measures.

## 3 Results

### 3.1 mTBI Induces Deficits in LTP by PID3

**FIGURE 2:**
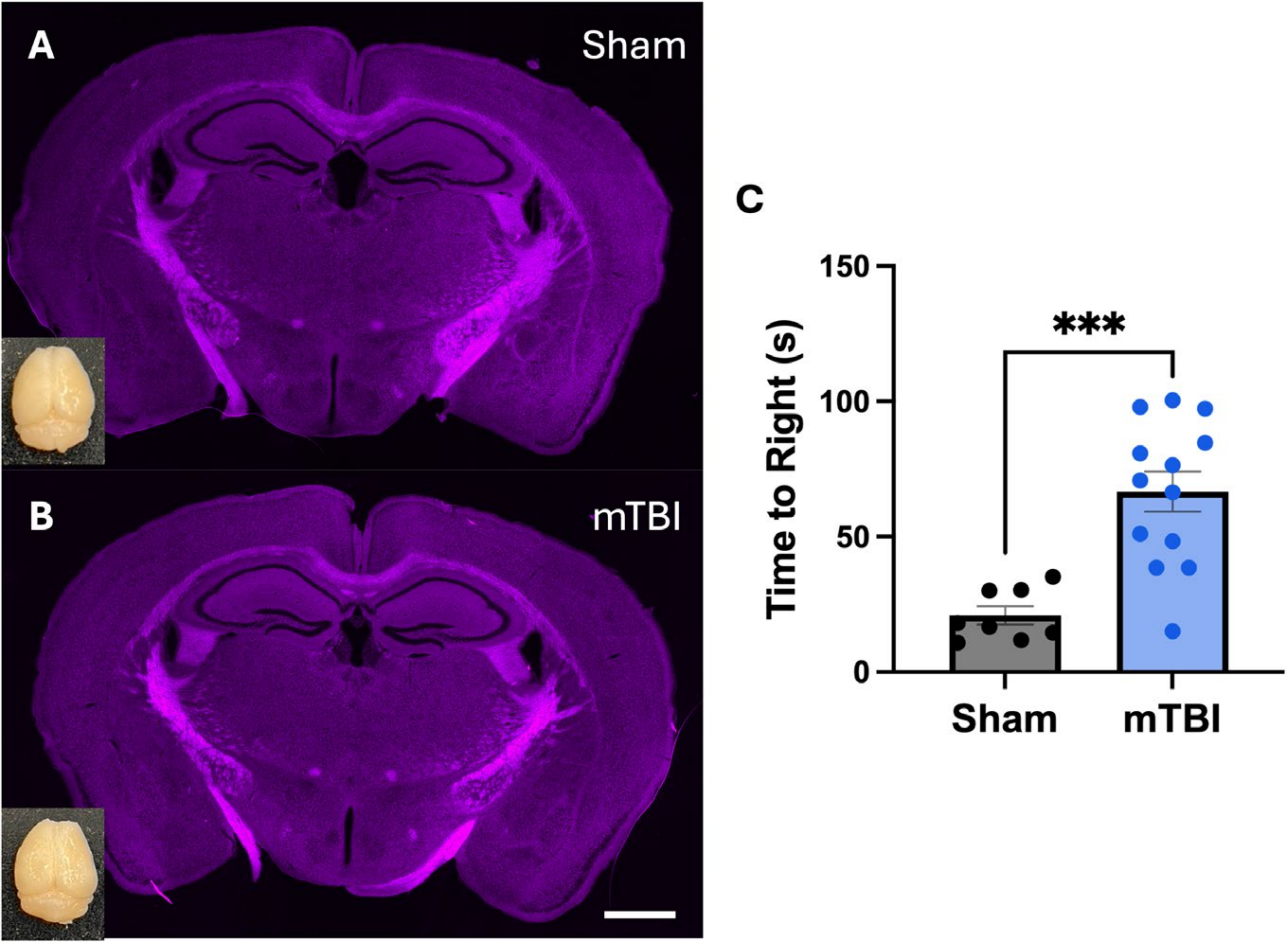
(A, B) Photomicrographs showing that the lateral impact model does not produce significant morphological damage to the brain. Scale bar is 1000 μm. (C) Following mTBI, adult mice display increased time‐to‐right compared to sham injuries. Error bars indicate SEM. *** indicates a statistically significant difference between sham and mTBI (*p* < 0.01).

**FIGURE 3:**
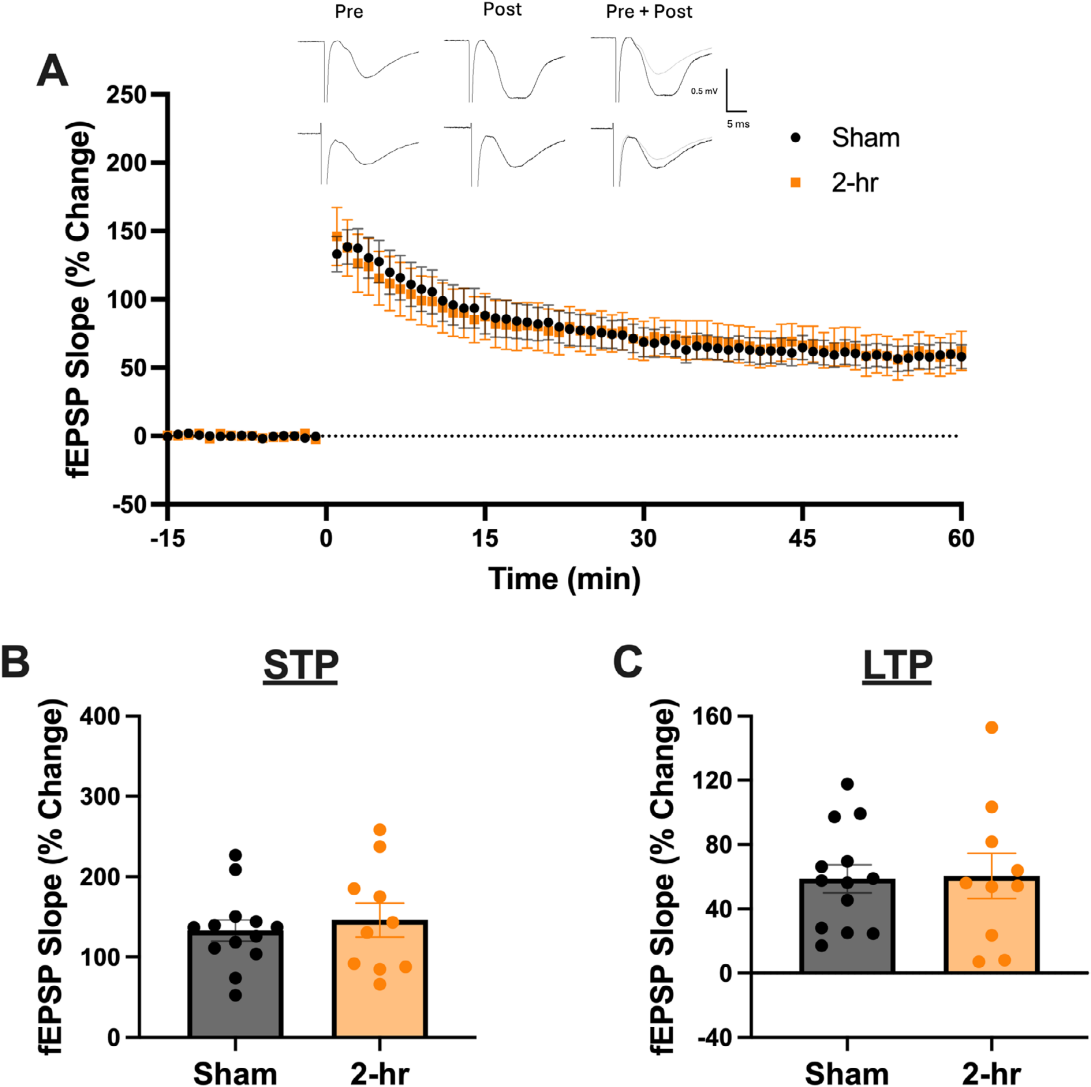
Mice do not display deficits in synaptic plasticity to 2‐h post injury in the medial perforant pathway of the DG. (A) Time course of synaptic plasticity experiments presented as a percentage of the baseline fEPSP. Baseline recordings in the presence of 5 μM BMI occurred from −15 to 0 min. Post conditioning responses (HFS; 4 × 100 Hz) recorded from 0 to 60 min. (B) There were no changes in short‐term potentiation (1 min following HFS). (C) There were no changes in long‐term potentiation (average of 55 to 60 min following HFS). Error bars indicate SEM.

**FIGURE 4:**
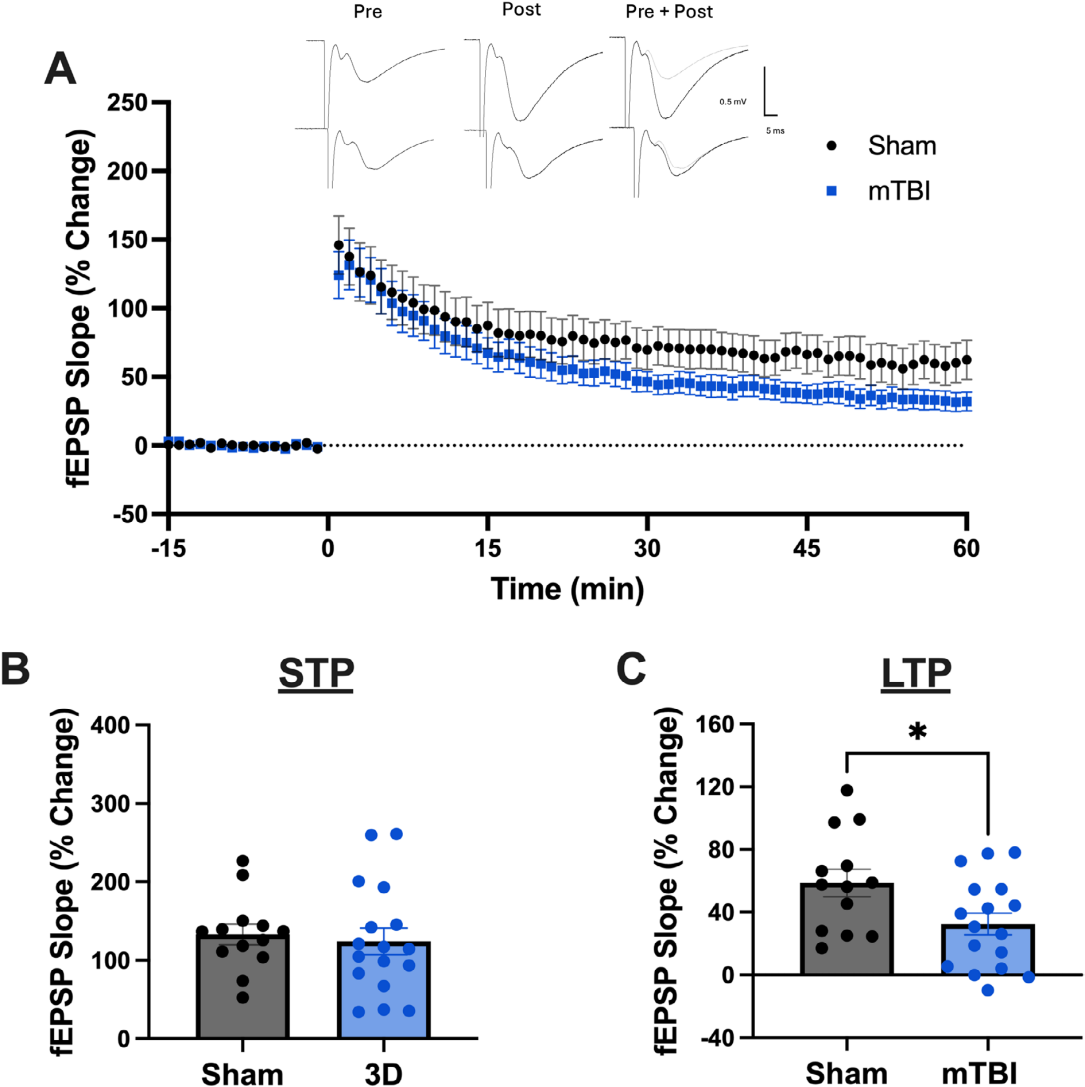
Mice display deficits in synaptic plasticity on PID3 in the medial perforant pathway of the DG. (A) Time course of synaptic plasticity experiments presented as a percentage of the baseline fEPSP. Baseline recordings in the presence of 5 μM BMI occurred from −15 to 0 min. Post conditioning responses (HFS; 4 × 100 Hz) recorded from 0 to 60 min. (B) There were no changes in short‐term potentiation (1 min following HFS). (C) Long‐term potentiation (average of 55 to 60 min following HFS) is decreased following mTBI. Error bars indicate SEM. * indicates a statistically significant difference between sham and mTBI (*p* < 0.05).

As an indicator that mice received an mTBI we used time‐to‐right as a measure of loss of consciousness (Mychasiuk et al. 2016; Wright et al. 2017; O'Reilly‐Fong et al. 2024). Here mice who received an mTBI displayed increased time‐to‐right compared to mice who received a sham injury (*t*(19) = 4.64, *p* = 0.01; Figure 2). Prior to incubation with AMS‐17 we needed to determine an optimal time for injury‐induced LTP deficits. Therefore, we prepared hippocampal slices at 2‐h and 3 days following mTBI. To determine changes in synaptic response we analyzed the average of the responses in the first minute following HFS (STP) and the average of 55 to 60 min following HFS (LTP). At 2‐h (Figure 3A) following injury there was no difference in the induction of STP (*t*(21) = 0.55, *p* = 0.59; Figure 3B) or LTP (*t*(21) = 0.47, *p* = 0.71; Figure 3C) as sham and mTBI animals displayed similar responses. Figure 4A displays the capacity for LTP at 3 days post‐injury which represents an acute injury timepoint during the peak of microglial reactivity following mTBI (Namjoshi et al. 2017; Simon et al. 2017). Similar to the 2‐h timepoint there were no differences between sham and mTBI animals in the ability to induce STP (*t*(28) = 0.39, *p* = 0.17; Figure 4B). However, the LTP was significantly reduced in mice who experienced an mTBI compared to animals who received sham injuries (*t*(28) = 2.39, *p* = 0.02; Figure 4C).

### 3.2 AMS‐17 Does Not Alter Presynaptic Neurotransmitter Release but Increases Basal Synaptic Transmission

**FIGURE 5:**
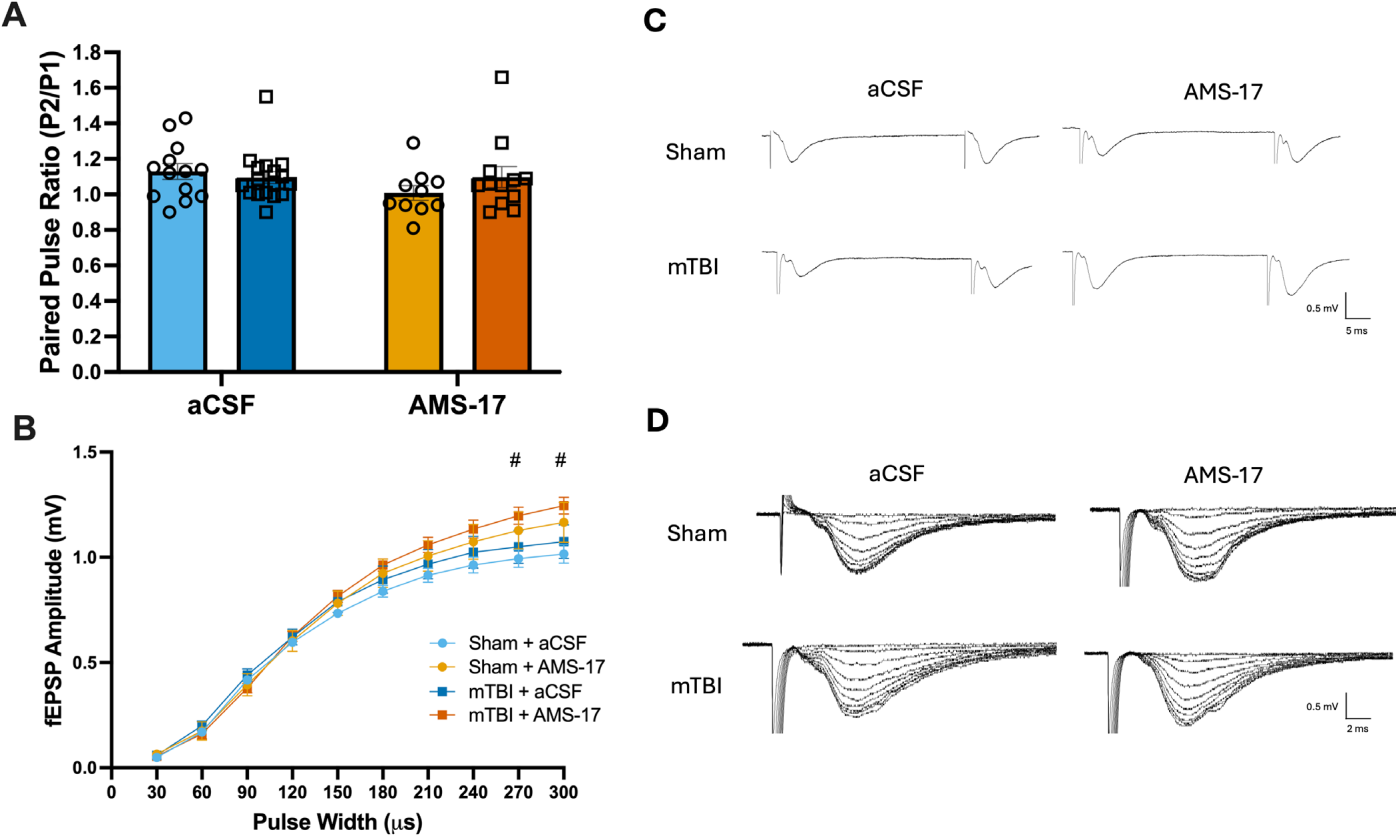
Incubation of hippocampal slices in AMS‐17 induces changes in basal synaptic transmission. (A) Paired‐pulse ratio (50 ms interpulse interval) remains unchanged from mTBI or incubation of hippocampal slices in AMS‐17. (B) Input–output curve displays enhanced basal synaptic transmission following incubation in AMS‐17. (C) Representative traces for paired‐pulse stimuli. (D) Representative traces for input–output curves. Error bars indicate SEM. # indicates a statistically significant difference between aCSF and AMS‐17 incubated hippocampal slices (*p* < 0.05).

Given the results at PID3 we used this timepoint to examine if inactivation of the NLRP3‐inflammasome had the potential to alleviate injury induced synaptic plasticity deficits. Hippocampal slices were incubated in 2.8 μM AMS‐17 in regular aCSF for 1–4 h, and then we assessed if incubation in AMS‐17 altered basic synaptic properties assessing presynaptic neurotransmitter release and basal synaptic transmission. A two‐way ANOVA for injury (sham; mTBI) and incubation drug (aCSF; AMS‐17) was performed to determine if this treatment produced any effects. To assess pre‐synaptic neurotransmitter release (Figure 5A,C), a pair of pulses (50 ms apart) was delivered and the paired‐pulse ratio was calculated (slope of pulse two divided by slope of pulse one). Incubation of slices (*F*(1,48) = 1.72, *p* = 0.20) and injury (*F*(1,48) = 0.35, *p* = 0.55) did not significantly impact pre‐synaptic neurotransmitter release on PID3. We also assessed whether the incubation in AMS‐17 would alter input–output curves (10 steps from 30 to 300 μs pulse widths; Figure 5B,D). Here we ran a repeated measures ANOVA for between subjects' factors injury (sham; mTBI) and incubation drug (aCSF; AMS‐17), and within‐subjects factor of pulse width. Mauchly's test of sphericity violated the assumption (*χ* ^2^(44) = 818.17, *p* = 0.001) so we adopted a Greenhouse–Geisser correction to our analysis. There were no significant main effects in the between‐subjects factors, but there was a significant interaction between pulse width and incubation compound (*F*(1.32, 63.10) = 5.56, *p* = 0.01). Post hoc analysis (with Bonferroni corrections) revealed that hippocampal slices that were incubated in AMS‐17 displayed an increase in the slope of the evoked fEPSP when compared to similar pulse widths as slices incubated in regular aCSF at 270 (*p* = 0.03) and 300 μs (*p* = 0.02).

### 3.3 AMS‐17 Ameliorates mTBI Induced Deficits in LTP in the MPP

**FIGURE 6:**
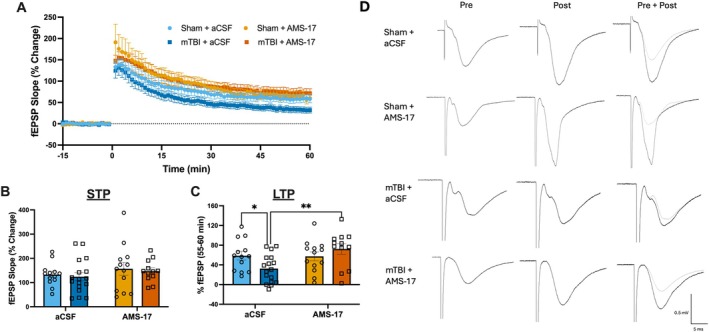
Incubation of hippocampal slices in AMS‐17 rescues deficits in LTP following mTBI in the medial perforant pathway of the DG. (A) Time course of synaptic plasticity experiments presented as a percentage of the baseline fEPSP. Baseline recordings in the presence of 5 μM BMI occurred from −15 to 0 min. Post conditioning responses (HFS; 4 × 100 Hz) recorded from 0 to 60 min. (B) There were no changes in short‐term potentiation (1 min following HFS). (C) Long‐term potentiation (average of 55 to 60 min following HFS) is decreased following mTBI. (D) Representative traces for baseline recordings (pre), long‐term potentiation (post), and an overlay of the two (pre + post). Error bars indicate SEM. * indicates a statistically significant difference (*p* < 0.05). ** indicates a statistically significant difference (*p* < 0.01).

Next, we assessed levels of STP and LTP in slices from sham or mTBI mice that were incubating in either regular aCSF or AMS‐17 (Figure 6A). Following HFS (4 × 100 Hz) incubation in AMS‐17 did not affect the ability to induce STP (0–1 min post‐HFS) in either sham or mTBI mice (Figure 6B). However, when we assessed the ability to induce LTP (55–60 min post‐HFS; Figure 6C,D), there was a significant main effect AMS‐17. Slices incubated in AMS‐17 displayed a greater capacity for LTP than slices that were incubated in regular aCSF (*F*(1,51) = 4.83, *p* = 0.03). There also was a significant injury by AMS‐17 interaction (*F*(1,51) = 5.56, *p* = 0.02). Post hoc testing revealed that mTBI animals displayed an enhanced capacity to induce LTP following incubation in AMS‐17 compared to mTBI animals with regular aCSF (*p* = 0.002). There were no changes in the capacity to induce LTP following sham injuries with or without incubation of AMS‐17 (*p* = 0.91), and there was no difference in LTP following AMS‐17 incubation for sham and mTBI (*p* = 0.25). This indicates that at PID3, inactivation of the NLPR3 inflammasome has the capacity to ameliorate mTBI induced deficits in LTP, bringing mTBI animals to sham levels.

## 4 Discussion

Neuroinflammation is a critical component of the pathophysiological response to mTBI. While an initial inflammatory response can facilitate recovery (Postolache et al. 2020), persistent and chronic inflammation can be detrimental (Simon et al. 2017). This may have direct implications for learning and memory processes, as cytokines play a critical role in modulating synaptic plasticity by altering NMDA and AMPA receptor expression at the synapse (Bourgognon and Cavanagh 2020). The purpose of this study was to determine if inactivation of the NLRP3 inflammasome with a novel inhibitor (AMS‐17) could ameliorate synaptic plasticity deficits following mTBI. Our findings indicate that LTP deficits appear to develop in line with the progression of inflammation, with deficits emerging around PID3. Furthermore, incubation of hippocampal slices in AMS‐17 successfully rescued LTP deficits following mTBI without affecting LTP induction in sham‐injured controls. These results suggest that targeting the NLRP3 inflammasome is a promising therapeutic strategy for helping to reduce learning and memory deficits following mTBI.

Previous studies have shown that mTBI induces an early proliferative response in the rodent hippocampus, with most proliferative cells associated with reactive microglia (Neale et al. 2023). Additionally, a single lateral impact mTBI has been associated with motor, anxiety, and cognitive impairments, as well as detectable mRNA markers of neuronal damage in the hippocampus (Mychasiuk et al. 2016; Wright et al. 2017). While not directly equivalent to the current paper, insights from repetitive models of mTBI can provide a useful framework to interpreting potential results. Previous use of the lateral impact model of repetitive mTBI has been linked to microglial reactivity, inflammatory responses, synaptic pruning, and deficits of learning and memory observed on PID5 (Eyolfson et al. 2020, 2022). Heightened neuroinflammation has been associated with impairments in LTP in the CA1 region of the hippocampus, yet the DG remains underrepresented in pre‐clinical research (Eyolfson, Suesser, et al. 2024). Long‐term potentiation deficits have been documented as early as PID1 and up to PID30 in the CA1 subregion following both single and repetitive mTBI (Almeida‐Suhett et al. 2015; Hu et al. 2022; Lecca et al. 2019; Liu et al. 2017; Sloley et al. 2021). In this study, we observed significant LTP deficits are evident at PID3 but not 2‐h following a single mTBI, aligning with previous findings that LTP deficits emerge more than 24 h after an injury (White et al. 2017). However, our study expands on these results demonstrating an effect in mice using the lateral impact model of mTBI, while previous studies utilized juvenile rats and the weight‐drop method. This is an important distinction as research has shown mTBI outcomes vary based on the direction of acceleration/deceleration forces (Mychasiuk et al. 2016). While cognitive deficits may persist well beyond PID3 (Zohar et al. 2003; Mouzon et al. 2012; Aungst et al. 2014), the use of this acute timepoint, during the height of inflammation and microglial reactivity (Namjoshi et al. 2017; Simon et al. 2017), to assess synaptic plasticity is important in our understanding of the mechanisms behind learning and memory. Detailing the synaptic response during this window may be important for the development of future therapeutic treatments for cognitive impairments.

Our findings indicate that mTBI does not affect basal synaptic properties, suggesting that LTP deficits are likely mediated by secondary injury cascades and NLRP3 inflammation. Given the well‐established role of NMDA and AMPA receptor signaling in LTP induction and maintenance, it is possible that cytokine‐mediated modulation of these receptors contributes to learning and memory impairments following injury. Indeed, cytokine expression plays a critical role in the facilitation and inhibition of synaptic plasticity (for reviews see (Bourgognon and Cavanagh 2020; Zipp et al. 2023)). For example, elevated IL‐1 β levels reduce NMDA and AMPA receptor availability in hippocampal synapses impairing non‐spatial memory, which can be recovered with administration of an IL‐1 receptor antagonist (Taoro‐González et al. 2019). These changes in NMDA and AMPA receptors associated with increased expression of mature IL‐1 β can also supress LTP in the hippocampus (Curran and O'Connor 2001). Beyond direct IL‐1 β effects, immune activation produces similar outcomes where, acute and chronic immune challenge with LPS produces deficits in LTP in the CA1 (York et al. 2021) and DG (Kelly et al. 2003). These deficits can be replicated (in the absence of LPS) with perfusion of IL‐1 β prior to HFS and recovered with an IL‐1 β receptor antagonist (York et al. 2021). Additionally, LPS‐induced LTP deficits can also be rescued with inflammasome inhibition (MCC950 or CAL‐0028) and caspase‐1 inhibition (Izumi et al. 2025). In the current study we report that inhibition of the NLRP3 inflammasome in hippocampal slices rescued LTP deficits associated with mTBI. The exact mechanism of recovery is unclear, but prior reports suggest that mTBI enhances NLRP3 activation, leading to caspase‐1‐ dependent maturation of IL‐1 β (Martinon et al. 2002). NMDA receptor phosphorylation and excess Ca^2+^ influx (Viviani et al. 2006; Dong et al. 2017; Jung et al. 2025), resulting in a downregulation of AMPAR receptor phosphorylation (Lai et al. 2006) and deficits in LTP. This indicates that targeting NLRP3‐mediatied inflammation may be a potential therapeutic for learning and memory deficits following TBI.

Preclinical studies have demonstrated a neuroprotective potential of NLRP3 inhibition in a number of neurological conditions. In a mouse model of Alzheimer's disease, the oral NLRP3 inflammasome inhibitor, OLT1177, improved learning and memory deficits in the Morris water maze, restored LTP deficits in Schaffer collateral synapses, and reduced microglial reactivity (Lonnemann et al. 2020). In the realm of TBI, Sun and colleagues showed that treatment with an IL‐1 receptor antagonist (1, 6, and 24‐h post injury, and daily for one‐week) recovered gene expression of reactive microglia and reduced cerebral edema following polytrauma (mTBI with concomitant tibial fracture). However, behavioral translation was inconsistent with no effect of treatment or injury at 12‐weeks post injury (Sun et al. 2017). Conversely, IL‐1RI−/− mice displayed recovered memory performance on the Barnes maze two‐weeks after moderate‐to‐severe TBI (Newell et al. 2018). These findings underscore the complex interactions between neuroinflammation and cognitive recovery, highlighting the need for further investigation into NLRP3 inhibition as a therapeutic strategy.

Although we did not directly examine synaptic morphology or density, it is possible that deficits in LTP are the result of loss of synapses in the hippocampus [for review, see (Jamjoom et al. 2021)]. Most evidence of altered synaptic proteins and spine density comes from moderate‐to‐severe models of TBI, where reductions in presynaptic (Synapsin‐I) and postsynaptic (PSD‐95) protein expression (Ansari et al. 2008a, 2008b) as well as decreased total number of synapses in the ipsilateral CA1 (Scheff et al. 2005) are seen as early as 48‐h post injury. Following fluid percussion injury (mild‐to‐moderate severity), increased apical and basal dendrite size were seen in the CA1 on PID7 (Schwarzbach et al. 2006), and these changes persisted through PID30 (Bray et al. 2022). In contrast, the effect of mTBI and repetitive mTBI appear to be more region dependent. After closed‐head mTBI, Urrutia‐Ruiz and colleagues reported a single mTBI caused reductions in ipsilateral and contralateral CA1 spine density as early as 10 days post injury, accompanied by an upregulation of immature spine morphology (Urrutia‐Ruiz et al. 2022). Following high‐frequency head impacts (five injuries per day for 6 days), mTBI resulted in LTP deficits in the CA1 on PID1, but this effect was not associated with changes in spine density, but instead to a decreased AMPA/NMDA ratio (Sloley et al. 2021). Repetitive mTBI have also been shown to produce increased spine density in the orbitofrontal cortex, prefrontal cortex, and nucleus accumbens (Christensen, Yamakawa, et al. 2020) but decreased spine density in the motor cortex (Eyolfson et al. 2022). The mechanisms of synapse loss following mTBI are not clear but could involve IL‐1 β, which has been shown to modulate synapse structure and number (Mishra et al. 2012).

The lateral impact model of mTBI has been implemented across a range of projectile velocities, typically 5 to 14 m/s in rats (Mychasiuk et al. 2016; Wright et al. 2017; Christensen, Eyolfson, et al. 2020; Sun et al. 2024; Zaini et al. 2025) and 5 to 10 m/s in mice (Eyolfson et al. 2020, 2022; O'Reilly‐Fong et al. 2024), in both single and repetitive injury paradigms. Previous work in mice has demonstrated that a single 5 m/s injury does not significantly increase time‐to‐right (Eyolfson et al. 2020; O'Reilly‐Fong et al. 2024). However, an increase in the number of injuries (3 to 5) or speed (7, 8, 9, 10 m/s) does significantly increase the time‐to‐right and motor deficits following mTBI (Eyolfson et al. 2020; O'Reilly‐Fong et al. 2024). In the present study, we assessed synaptic plasticity deficits following a single concussive injury delivered at 7 m/s. Consistent with prior work, mice who received an mTBI displayed increased time‐to‐right latencies compared to sham animals (O'Reilly‐Fong et al. 2024). While beyond the scope of this paper, these converging results seem to highlight that repetitive injuries during the window of cerebral vulnerability (Longhi et al. 2005) induce significant pathophysiological responses that may not be captured by single‐injury models alone.

In conclusion, this work provides preliminary evidence that therapies focused on the inactivation of the NLPR3‐inflammasome may be a valuable strategy to mitigate synaptic plasticity deficits following mTBI. It should be noted that we only tested a single concentration of AMS‐17 at a single timepoint, so future studies should extend this work to later timeframes (e.g., PID7, PID30) commonly associated with deficits in synaptic plasticity (White et al. 2017; Sloley et al. 2021; Hu et al. 2022). There is also a need to create a drug response curve to determine the optimal therapeutic window for AMS‐17. Further rigorous testing will also be required to determine if AMS‐17 has any adverse effects like other NLRP3 inhibitors currently in use. Although we did not quantify cognitive impairment, implementing a test such as the Morris Water Maze or Radial Arm Maze to validate the rescue of a functional deficit could strengthen the literature of efficacy of AMS‐17 as an mTBI treatment. Given the current design we are unable to determine the mechanism of action rescuing LTP deficits with AMS‐17. It will be important for future studies to determine if microglial reactivity or inflammasome protein or mRNA expression is altered at PID3. Additionally, it will be important to determine if changes in LTP are due to altered inflammasome activation, enhanced IL‐1 β signaling, and affecting NMDAR expression at the synapse or changes in synapse numbers following injury. Closed‐head models of mTBI are noted to produce sex‐differences in behavioral and pathophysiological responses in both mice and rats. However, research is limited within synaptic plasticity deficits following mTBI. This is a limitation of the current study as we were not powered to separate the response of females and males, and it will be an important distinction to make in future studies. Finally, it will be important to replicate these results following repetitive mTBI (r‐mTBI) as those who experience a single mTBI are at increased risk to receiving future mTBIs, with exacerbated pathophysiological responses. The goal of this pilot study and subsequent follow up experiments will be to determine if in vivo administration of AMS‐17 can reduce the inflammatory response, microglial reactivity, and behavioral deficits in mice.

## Author Contributions

**Eric Eyolfson:** conceptualization, methodology, investigation, formal analysis, writing and editing manuscript. **Luis Bettio:** investigation and editing of manuscript. **Justin Brand:** investigation, formal analysis, and editing of the manuscript. **Naveen Kumar Gupta:** compound development and editing of manuscript. **Emily Hamer:** compound development and editing of manuscript. **Ryan Salas:** compound development and editing of manuscript. **Amol Kulkarni:** conceptualization, methodology, development of compound, reviewing and editing, supervision. **Brian R. Christie:** conceptualization, methodology, investigation, reviewing and editing, supervision.

## Funding

This work was supported by the Natural Sciences and Engineering Research Council of Canada (RGPIN‐2019‐06104) and the National Institutes of Health (7R21NS129478‐03, 1R21DA059788‐01).

## Conflicts of Interest

The authors declare no conflicts of interest.

## Data Availability

The data that support the findings of this study are available on request from the corresponding author. The data are not publicly available due to privacy or ethical restrictions.
